# Using a logical model to predict the growth of yeast

**DOI:** 10.1186/1471-2105-9-97

**Published:** 2008-02-12

**Authors:** KE Whelan, RD King

**Affiliations:** 1 Department of Computer Science, Aberystwyth University, Aberystwyth, Wales, UK

## Abstract

**Background:**

A logical model of the known metabolic processes in *S. cerevisiae *was constructed from iFF708, an existing Flux Balance Analysis (FBA) model, and augmented with information from the KEGG online pathway database. The use of predicate logic as the knowledge representation for modelling enables an explicit representation of the structure of the metabolic network, and enables logical inference techniques to be used for model identification/improvement.

**Results:**

Compared to the FBA model, the logical model has information on an additional 263 putative genes and 247 additional reactions. The correctness of this model was evaluated by comparison with iND750 (an updated FBA model closely related to iFF708) by evaluating the performance of both models on predicting empirical minimal medium growth data/essential gene listings.

**Conclusion:**

ROC analysis and other statistical studies revealed that use of the simpler logical form and larger coverage results in no significant degradation of performance compared to iND750.

## Background

### In Silico modelling of cellular processes

This paper describes the construction and application of a logical model of known metabolic processes in bakers' yeast (*Saccharomyces cerevisiae*). The model outlines the relationships between Open Reading Frames (ORFs), enzymes and reactions comprising the metabolic pathways in yeast, presenting the knowledge as facts in a restricted form of First Order Logic (FOL). This representation allows the model to behave as a deductive (or relational) database, as well as a model for the study of metabolic behaviour.

A focus of our previous research has been the development and implementation of an automated system for the design, execution and interpretation of wet lab auxotrophic experiments: the Robot Scientist [1,2]. In its first incarnation the scope of the Robot Scientist was limited to rediscovery of knowledge from a single metabolic pathway in yeast (the Aromatic Amino Acid (AAA) biosynthesis pathway), where a logical model of this pathway [3] acted as background knowledge from which the Robot Scientist was able to generate hypotheses. The logical model presented here, "aber", is an updated and expanded version of this limited model (the name is derived from "Aberystwyth" – where the model was constructed). The model has been expanded to include most of what is known about yeast metabolism. The model has also been updated to a more explicitly relational data structure; replacing the single relation that included enzyme and metabolite details with separate relations, enabling a more natural and versatile knowledge representation.

To evaluate the correctness of the model we have used it to predict the results of empirical growth experiments of knockout mutants growing on defined minimal medium [4]. Although our model has been designed to be able to generate growth predictions on defined growth media, the model contains many essential genes, therefore a list of essential genes generated by the same gene-deletion study was used in conjunction with the minimal medium growth results to more accurately reflect the predictions of the model. An assessment of the ability of the model to correctly predict gene essentiality was also performed; in this case the predictions of growth were compared to the list of essential genes alone, with no reference to the minimal medium experimental data. ROC analysis was used to compare this logical model to *iND750 *[5], a state-of-the-art Flux Balance Analysis (FBA) model [5,6] (iND750 reflects a naming scheme for systems biology models, *i *refers to a *in silico *model, *ND *reflects the creators of the model (Natalie Duarte) and *750 *represents the number of genes included in the model). Both models have also been compared with the predicted growth outcomes generated by a simple majority class classifier, as well as the probability that prediction success was purely random.

### Systems biology and the modelling of biochemical networks

Systems Biology [7-10] represents a shift towards a synergistic approach to whole cell modelling, with the concentration on the interactions of many inter-related components rather than the behaviour of the individual components. Advances in mathematics and computer science have led to the development of diverse techniques and formalisms allowing the *in silico *modelling of these cell systems. All computer models represent varying degrees of abstraction from the observable phenomena they represent, from coarse large scale models that capture only essential interactions and components of the system e.g. KEGG [11,12] and EcoCyc [13], to higher fidelity representations of detailed functioning and interactions of a smaller set of components [14].

There are two main groups of modelling techniques used to represent metabolic networks:

1) Quantitative methods that aim to capture the changes in the quantities of metabolites and enzymes etc. by representing cellular processes, e.g. reaction kinetics, in detail

2) Qualitative methods that aim simply model the presence of interactions, e.g. reactions are modelled as chemical transformations, with no representation of reaction kinetics

The two most important quantitative methods are ODE (Ordinary Differential Equations) and FBA (Metabolic Flux Balance Analysis). ODEs are the most established modelling representation in science. In using ODEs to model metabolism the concentration of each metabolite is calculated by a single ODE encapsulating all the reactions where the metabolite is synthesised or consumed, with fluxes determining the transformations to and from other metabolites in the network. The use of ODE models is the main technique of the quantitative sciences. There are also now a number of specialised ODE modelling packages for metabolism, e.g. Gepasi/Copasi [15-17] and e-Cell [18]. Despite this, the current application of ODEs to modelling large-scale metabolism has a number of serious problems: we do not know, nor are likely to know soon, all the necessary parameters that such models demand; and there can be numerical analysis problems in solving ODE metabolic models, resulting in quantitative and qualitative differences between simulators.

Flux Balance Analysis (FBA) [5,6,19-21] is currently the most common approach to quantitatively modelling metabolism. Standard FBA assumes a steady state model of cell metabolism; although more recent developments in FBA [20] have extended this to dynamic flux balance analysis that is capable of modelling cells with some state change. Cell reactions are modelled by two matrices: one corresponding to the stoichiometry of the reactions, the other containing the fluxes for the reactions. Typically many of these fluxes remain unknown and experimental measurement of the ranges for a small number of "control" fluxes and linear/nonlinear programming is used to determine the values of the unknown fluxes, based on an assumption of optimality. FBA models of the metabolism of a number of organisms exist, e.g. *S. cerevisiae *[5,6] and *E. coli *[19,20] and recently the human metabolic network [21]. The steady state assumption (relaxed for dynamic FBA however) and the inaccuracies of the unknown fluxes can lead to inaccuracies in the overall simulation [22]. However, increased experimental evidence can decrease these inaccuracies.

A number of flux based alternatives to FBA have been developed that address the changes in fluxes that occur after major environmental or other perturbations such as gene knockouts: MOMA (Minimisation of Metabolic Adjustment) [23] and ROOM (Regulatory on/off minimisation) [24]. Both make use of distance measures to determine a point in flux space that is closest to the wild type flux distribution, in keeping with a homeostasis hypothesis. MOMA minimises the changes to each individual flux, thereby the overall network is as similar as possible to the wild type. In contrast, ROOM minimises the number of significant flux changes, thereby better approximating how short alternative pathways allow redundancy in metabolic networks. In a predictive study using lethal/non-lethal knockouts in *E. coli*, ROOM had an accuracy of 85%, which is comparable to the standard FBA approach. Elementary mode analysis [25] determines the set of smallest sub-networks of a larger metabolic network that still allow a metabolic steady state to be reached. Each elementary mode represents an alternative that the organism may use in conditions of perturbation. Stelling *et al *[25] use elementary-mode analysis to determine lethal/non-lethal gene deletions in the central carbon metabolism of *E. coli*, where lethal mutants mostly have an empty set of elementary modes, and non-lethal mutants have at least one elementary mode with an overall positive growth rate. Stelling *et al *[25] observed a 90% correct prediction rate for the 90 knockouts they studied. However there can be a large number of elementary modes for even a moderately large metabolic network, indicating that there may be problems scaling this approach to whole metabolism networks.

Logical and Graph (LG) based models [1,11-13] are the commonest qualitative representations for modelling metabolism. Graph based models are used in metabolic databases e.g. KEGG [11,12], EcoCyc/MetaCyc [13]. Metabolic pathways are represented explicitly, each metabolite is a node in the graph and edges represent the chemical transformations found in the reactions comprising the pathway. Edges are further annotated by the enzyme(s) that catalyse the reactions, and these are in turn are related to the gene(s) that encode the enzymes. Lemke *et al *[26,27] have developed a graph-based model of the metabolic network of *E. coli *and have used it to analyse how much damage the absence of each enzyme causes to the metabolic network. Metabolic damage is defined as the number of metabolites that can no longer be produced by the organism. They show that only 9% of enzymes prevent production of 5 or more metabolites, but that more than 50% of essential enzymes are to be found in this group.

Logical models may use computationally efficient forms of both propositional and FOL (Prolog) as their representation language [28]. As in graph models, the reaction network is represented by a series of metabolite nodes and chemical transformation arcs, however the increased expressive power of logic can allow more accurate representations of the relationships between the genes, enzymes and gene products used as annotations to the reactions, as well as various cellular compartments. For example, only enzyme names are used to annotate the reaction arcs in Lemke *et al *[6,27], unlike ORF and EC number for the aber model. Metabolic modelling is done by keeping a tally of the metabolites added to the cell by each reaction (see section 5 for more detail).

The BIOCHAM system [29] is a dedicated biochemical reasoning engine that uses a rule based temporal logic language to model and query all of the possible behaviours of a given biochemical model, and the MAPK signal transduction cascades have been used as an example. Random Boolean networks [30,31] have also been used to model 106 of the genes comprising the yeast transcriptional network. Random Boolean are useful for modelling systems where interactions are not known beforehand. Kauffman *et al *[31] used this process to identify those networks that were most stable, thereby representing rules of biological relevance to the regulation of gene transcription. In general the graph models are equivalent to propositional logic models and can adequately represent metabolic reactions as nodes and arcs. However this representation is not expressive enough to adequately represent the relationships between ORF/enzymes etc. that control the reactions. The increased expressivity of FOL is required for this additional complexity.

The use of qualitative reasoning (QR) [32] is an intermediate representation between quantitative and logical models, e.g. King *et al *used QR to model the Glycolysis pathway [33]. Rather than presence/absence of a metabolite in the cell as in the coarse representation of logical/graph models, or the quantitative concentration as in ODE models, QR models record the qualitative state of each metabolite, e.g. whether the concentration is increasing, decreasing or constant. Qualitative differential equations [33] are used to calculate the change in quantity of the metabolites and enzymes, with a FOL representation of the metabolite and enzyme components of the system. A common task in QR is to generate the complete envisionment of the system, i.e. all of the possible qualitative states that can be derived from an initial starting state. This can be a computationally expensive task, King *et al *found 27,254 possible states for the Glycolysis model.

## Results

To test the utility of our "aber" model we compared it with the iND750 FBA model, and a majority classifier that assigns each prediction to the most commonly occurring classification (in this case continued growth of the strains). Although iND750 and the aber model have both been constructed from iFF708 [6], subsequent development has led to a divergence of the number of ORFs for which there are experimental results in Giaever *et al *[4]: iND750 makes predictions for 681 ORFs, while the aber model makes predictions for 940, with 641 predictions shared by both models. Evaluation of the performance of the models examined the shared genes as well as the total number of genes in each model, so that the effect of adding newly categorised ORFs to the original set can also be examined.

Two different cases of gene knockout sensitivity on MMD+ura+hist+leu were considered, 1) genes found to be significantly sensitive after 5 generations and 2) genes found to be significantly sensitive after 5 generations that remain significantly sensitive after 15 generations. As for iND750, the metabolic network and the starting media definitions used by the aber model were altered to reflect the additional requirements for uracil, histidine and leucine. An additional comparison was also made with the iND750 results where a preprocessing step was used to identify borderline cases of retarded growth in the sensitive/refractory data. This preprocessing step used all of the sensitivity results for each ORF, ignoring any refractory scores for ORFs, therefore all of the sensitivity data is used rather than focusing only on ORFs found to be sensitive in both Giaever experiment sets (The iND750 results may include ORFs not found to be sensitive in both sets). The imbalance of prediction counts also results in a shortfall of experiment results from the iND750 preprocessing step (comparison set C in Table 1). In cases where no experimental results from comparison set C were available, results from set B were used. Definitions of the 3 sets of experimentally derived growth and no growth outcomes are given in Table 1.

**Table 1:** Comparison set definitions for comparing model predictions to experimental results using MMD defined growth medium

**Prediction/Experiment Comparison Set**	***Growth *Outcome**	***No Growth *Outcome**
**A**	**NOT ***essential ***AND NOT ***significantly sensitive *after 5 generations in both experimental repeats	*essential ***OR ***significantly sensitive *after 5 generations in both experimental repeats
**B**	**NOT ***essential ***AND NOT ***significantly sensitive *after both 5 and 15 generations in both experimental repeats	*essential ***OR ***significantly sensitive *after both 5 and 15 generations in both experimental repeats
**C**	**NOT ***essential ***AND NOT ***sensitive by iND750 preprocessing*	*essential ***OR ***sensitive by iND750 preprocessing*

Although the logical model uses MMD+ura+hist+leu as its set of starting compounds, comparison with the essential gene list is valid because knockouts that do not grow on rich YPD will also almost certainly not grow on a simple defined medium. Analysis of the model comparisons made use of performance metrics and statistical tests that evaluated the significance of any observed difference in model performance. These are described below, followed by a discussion of the gene essentiality findings and the results of the minimal medium growth study

### Skewed performance metrics and significance tests

In an analysis of the use of ROC space Flach [34] defines a number of metrics that use a skew ratio (*c *in Table 2) to allow for bias introduced by classes of dissimilar size (as is the case for the model predictions and the experimental results.) These metrics can be used to both measure a classifier's performance and as conditions for evaluation of rules/tree nodes etc. as classifiers are constructed. The metrics are *skewed accuracy *and *skewed precision*. Table 2 shows the formulas for these two metrics as well as formulas for metrics representing intermediate steps. In this table TP represents the number of true positive predictions, TN the number of true negatives, and FP and FN the number of true and false positives and negatives respectively. POS corresponds to the total number of positive predictions and NEG, the total number of negative predictions.

**Table 2:** Formulas used to calculate Model Validation metrics

***Metric***	***Formula***
*tpr (true positive rate)*	$tpr=\frac{TP}{POS}$
*fpr (false positive rate)*	$fpr=\frac{FP}{NEG}$
*rfp (relative frequency of positives)*	$rfp=\frac{POS}{\left(POS+NEG\right)}$
*C (class ratio or skew ratio)*	$c=\frac{NEG}{POS}$
*sk_acc (accuracy)*	$sk\_acc=tpr+c\frac{\left(\text{1}-fpr\right)}{\text{1}}+c$
*sk_prec (precision)*	$sk\_prec=\frac{tpr}{tpr+c\cdot fpr}$

A McNemar χ^2 ^test was used to compare the performance of the aber model, iND750 and the majority classifier on each of the 3 comparison sets. All three sets of ORF sources (aber, iND750 and shared) were used for comparison of the aber model and iND750. The shared ORFs and aber ORF sources were used for the comparison of the aber model and the majority classifier. The iND750 ORFs and shared ORFs sources were used for the comparison of iND750 and the majority classifier. The McNemar test analyses the proportions of mistakes for predictions common to both models. To enable comparison of equivalent numbers the majority class was used to obtain predictions for ORFs not found in either model (i.e. the 40 ORFs from iND750 not found in the aber model and the 299 ORFs in the aber model not found in iND750). Significance for the McNemar tests was determined for p > 0.05 and the direction of the test result was collected (i.e. which model outperforms the other if a significant difference is observed). The following equation defines the McNemar test used, where b is the number of predictions found to be correct by model 1 and incorrect by model 2 and c is the converse (incorrect by model 2 and correct by model 1). A Cumulative binomial test was also used to determine the probability of generating the predictions of the aber model by chance.

$$\chi^{\text{2}}=\frac{{\left(\text{abs}\left(b-c\right)-\text{1}\right)}^{\text{2}}}{b+c}$$

### Predicting gene essentiality

We use the ability of a metabolic network models to predict the essentiality of a gene as a measure of model quality. Gene essentiality results have been presented for iFF708, the prime source of knowledge for the aber model and the precursor to iND750, to which the aber model is compared. Förster *et al *[35] report a predictive accuracy of 85% for gene essentiality for iFF708. A model's prediction can be tested by comparison with the empirical "wet" experiment of growing the knockout strain. The growth (*phenotype*) of a knockout strain depends on both its *genotype *(what gene(s) have been removed) and the *environment*. To use a model of metabolism to predict gene essentiality, it is therefore necessary to know the composition of the growth medium (the environment). This makes the use of "wet" results using the most common growth medium for yeast, YPD, problematic because its exact composition is undefined. This is why we have focused on using the Giaever *et al *data [4] for growth on a defined minimal medium.

Table 3 shows the skewed accuracy and skewed precision results for the aber model, iND750 and the majority classifier (maj) for the 3 sets of genes described above and Table 4 presents the results of McNemar tests to determine the significance of any difference in the three models. These results show very little variation in performance, with iND750 scoring slightly higher in terms of accuracy on the ORFs corresponding to its own source set. The McNemar tests also show no significant difference in performance between all three models, for all three sets of ORFs. This is a reflection of the small number of essential vs non-essential ORFs (18.4 % of the shared ORFs and those from the aber model are essential; 17.5% of the iND750 ORFs are essential). Indeed the slightly smaller proportion of essential ORFs in the iND750 set may account for the apparent increase in accuracy for IND750. The accuracy results for the aber model and iND750 are slightly poorer than the results for iFF708, indicating that expansion of both models has slightly decreased the ability to predict essential genes.

**Table 3:** Performance results for gene essentiality predictions

**ORF Source**	**Aber**	**Shared**	**iND750**	**Shared**	**Aber**	**iND750**	**Shared**
**Model**	**Aber**	**Aber**	**iND750**	**iND750**	**maj**	**maj**	**maj**
**Num ORFs**	**940**	**641**	**681**	**641**	**940**	**681**	**641**
**Skewed Accuracy**	0.81	0.80	0.82	0.80	0.82	0.83	0.82
**Skewed Precision**	0.83	0.86	0.84	0.85	0.82	0.83	0.82

**Table 4:** Results of McNemar significance tests for gene essentiality

**Comparison**		**Aber ORFs (940)**	**iND750 ORFs (681)**	**Shared ORFs (641)**
**Aber Model vs iND750**	**result**	1.19	2.93	2.64
	**significance**	no	no	no
	**direction**			
**Aber Model vs Majority Classifier**	**result**	0.31	NA	0
	**significance**	no		no
	**direction**			
**iND750 vs Majority Classifier**	**result**	NA	2.56	2.31
	**significance**		no	no
	**direction**			

### Comparing predicted experiment outcomes to growth outcomes obtained by a whole genome gene-deletion study and performance of iND750

Table 5, containing results for growth predictions on the defined medium, shows that all three classifiers have similar values for the performance statistics indicating similar performance for the logical model, iND750 and the majority classifier on all of the comparison sets; however there is a slight decrease in performance on comparison sets B and C. The McNemar test results (Tables 6, 7 and 8) indicate that there is no significant difference in the predictive accuracy of the aber model and iND750 for all ORF sources and all comparison sets, and that both models perform significantly better than the majority classifier on all but the most stringent definitions of growth/no growth. This is the case where the majority class is at its largest (82% or 83%) and where neither predictive model performs better. *This indicates that use of the more complicated formalism of FBA does not improve the prediction of healthy/retarded growth compared to logical modelling. *However this observation must be tempered by the fact that a fully direct comparison between the FBA prediction technique and the logical model prediction technique has not been possible, because the network corresponding to the shared ORFs is incomplete. The results from the McNemar tests also indicate that expanding the coverage of the model by adding information from KEGG has not significantly compromised the performance of the component of the model originating from iFF708. Indeed, it has been possible to increase the coverage of the metabolic network by around 30% without a significant degradation of performance. There was also no significant difference between both the aber model and iND750 on comparison set B – the more stringent definition of sensitive growth. This is partly because of the skewed nature of the experimental results (the skew ratio C varies between 0.22 and 0.34 for all result sets and ORF source combinations) and partly because of the generally high false positive rates (variation from 0.55 to 0.86), therefore both iND750 and the aber model make more incorrect predictions for growth.

**Table 5:** Values for metrics used to validate models for all experimental comparisons using defined medium

	**ORF Source**	**Aber**	**Shared**	**iND750**	**Shared**	**Aber**	**iND750**	**Shared**
	
	**Model**	**Aber**	**Aber**	**iND750**	**iNd750**	**maj**	**maj**	**maj**
	
	**No ORFs**	**940**	**641**	**681**	**641**	**940**	**681**	**641**
**Skewed Accuracy**	**Exp A**	0.79	0.79	0.80	0.80	0.77	0.77	0.76
**Skewed Precision**	**Exp A**	0.80	0.80	0.83	0.82	0.77	0.77	0.76
**Skewed Accuracy**	**Exp B**	0.81	0.82	0.80	0.79	0.82	0.83	0.82
**Skewed Precision**	**Exp B**	0.83	0.84	0.86	0.85	0.82	0.83	0.82
**Skewed Accuracy**	**Exp C**	0.83	0.85	0.84	0.83	0.82	0.83	0.82
**Skewed Precision**	**Exp C**	0.83	0.85	0.85	0.84	0.82	0.83	0.82

**Table 6:** McNemar test results for the aber model vs iND750

**Comparison Set**		**Aber ORFs (940)**	**iND750 ORFs (681)**	**Shared ORFs (641)**
**A**	**result**	0.72	0.60	0.14
	**significance**	no	no	no
	**direction**			
**B**	**result**	1.19	2.93	2.64
	**significance**	no	no	no
	**direction**			
**C**	**result**	0.25	1.05	1.09
	**significance**	no	no	no
	**direction**			

**Table 7:** McNemar tests results for the aber model vs the majority classifier

**Comparsion Set**		**Aber ORFs (940)**	**Shared ORFs (641)**
**A**	**result**	5.02	8.51
	**significance**	yes	yes
	**direction**	Aber	Aber
**B**	**result**	0.31	0
	**significance**	no	no
	**direction**		
**C**	**result**	5.94	11.17
	**significance**	yes	yes
	**direction**	Aber	Aber

**Table 8:** McNemar results for iND750 vs the majority classifier

**Comparison Set**		**iND750 ORFs (681)**	**Shared ORFs (641)**
**A**	**result**	5.01	5.69
	**significance**	yes	yes
	**direction**	iND750	iND750
**B**	**result**	2.56	2.64
	**significance**	no	no
	**direction**		
**C**	**result**	34.38	134.31
	**significance**	yes	yes
	**direction**	iND750	iND750

### Estimating the probability of chance occurrence of model predictions

A cumulative binomial test was used to evaluate the probability that the predictions made by the logical model could have been made by chance. This test calculates the significance of deviations from the binomial distribution of observations falling into two categories (predictions of growth and no growth), assuming a random binomial model that assigns genes to essential/retarded growth and non essential/healthy growth categories by chance. The probability is calculated for the range *r..n*, where *r = 889*: the number of predictions for growth and *n *is the total number of predictions made (growth + no growth: *940*):

$$P{\left(X\geq r\right)}_{bin}=\sum_{i=r}^{n}\left(\frac{n!}{\left(n-i\right)!\cdot i!}\cdot p^{i}\cdot q^{q-i}\right)$$

where *p = 0.82*, the proportion of ORFs that result in experimental growth and *q = 1 - p *(the proportion of essential/retarded growth genes). This test resulted in a probability, *P*(*X *≥ 889)_*bin *_= 8.81 × 10^-32^: a very low probability that the predictions were generated by chance.

## Discussion

Comparison of our logical model with iND750 demonstrated that there is no significant difference in the performance of any of the models on the prediction of gene essentiality. A significant increase in performance was observed for both the aber model and iND750 w.r.t the majority classifier on predicting growth outcome on defined growth medium, for all except the most stringent definition of growth from Giaever *et al *[4]. There was also no significant difference between the aber model and iND750 in the defined growth medium study. The gene essentiality study indicates that both models are equally poor at predicting essential genes, indeed neither is an improvement on the simple majority classifier. As is the case for the minimal medium experimental data, the essential gene data is highly skewed, reflecting the amount of redundancy in the yeast genome. The improvement in performance of both models w.r.t the defined medium study is a result of the precise definition of the medium components (only approximations to undefined media are possible). The similarity of results for the aber model and iND750 indicate that the additional complexity of FBA is not required for the prediction of essential genes or the determination of growth/retarded growth on defined media. However the logical model is currently restricted to binary growth/no growth predictions and has no capability to predict growth rates unlike FBA based techniques. It is a case of horses for courses. The logical model presented here is a useful addition to the currently existing models with an appropriate mix of expressive representation of metabolic concepts and a simple mechanism for determining predictive outcomes.

The high number of inaccuracies in the prediction of growth for all models indicates that much important information remains to be discovered about yeast metabolism.

### Using the logical model as background knowledge for active learning of hypotheses

**Figure 1:**
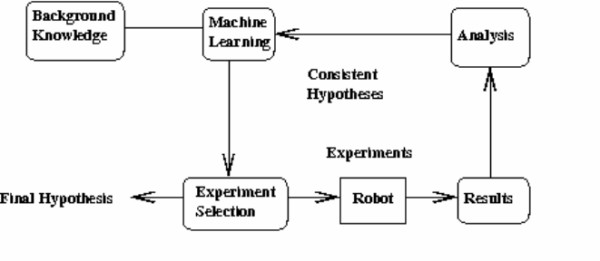
Active Learning for Scientific Discovery.

A major motivation for our use of a logical formalism is that it enables the exploitation of techniques from the large research area of Inductive Logic Programming (ILP) and related first-order learning methods [36]. This is not possible with other formalisms such as FBA. In Previous work [1], a precursor of the aber logical model was used as a background theory in a (re)discovery task involving the function of ORFs from a single pathway (AAA Biosynthesis) in Yeast. The task was implemented as an active learning loop, a machine learning technique based on abductive logic programming that mirrors the hypothetico-deductive process of hypothesis formation in scientific discovery. In active learning a classifier is given a set of training examples incrementally, so that the most informative example (given a current hypothesis set) can be chosen at each iteration of the loop. In scientific discovery training examples correspond to the outcome of experiments carried out automatically by a lab robot, and the active selection of a training example corresponds to choosing the experiment most likely to refute the largest number of hypotheses, given the outcome of the experiment (growth or no growth). Figure 1 illustrates the how the active learning loop was applied to the functional genomics discovery task.

**Figure 2:**
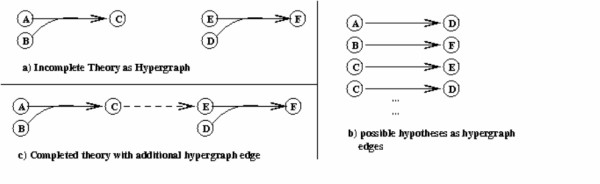
Abductive Inference for Graph Completion.

The formation of hypotheses from the outcomes of the selected experiments was performed by a limited form of abductive theory completion [3], performed by PROGOL, an ILP [36] program that performs both abductive and inductive machine learning in a restricted form of FOL. In this task the logical model formed the incomplete background knowledge theory, to which the final hypothesis is added to "complete" or improve the theory, therefore adding to the current biological knowledge of yeast functional genomics. The completion of the theory is analogous to identifying a new edge to be added to the metabolic network, as well as a new ORF annotation for that edge. This is described in Figure 2.

The use of this restricted form of FOL allows all components of the scientific discovery task to be described using the same knowledge representation, indeed the complexity of the entities and relationships required for accurate description of enzymes, reactions, experiments, and hypotheses etc can only be expressed by FOL or an equivalently expressive knowledge representation (e.g description logics, OWL). Abduction of hypotheses relating ORFs, enzymes and reactions also requires that the machine learning program has FOL as it's representation language, hence the use of programs from ILP. The Robot Scientist concept therefore represents a close integration of logical inference techniques used for scientific discovery and an automated laboratory that can perform experiments. The previous work on the AAA Biosynthesis pathway was a proof of principle study. Recently the original robot hardware has been replaced by a state-of-the-art automated laboratory that substantially increases the throughput of the Robot Scientist [37]

## Conclusion

This paper presents a logic based model of the metabolic processes in *S. cerevisiae*: "aber". The core of the aber model is the FBA model iFF708 [6]. Added to this core are an additional 263 ORFs, and also includes an additional 247 reactions taken from KEGG [11,12]. The aber model has been designed to enable automated reasoning about yeast metabolism. Use of the model to predict the essentiality of genes in knockout studies provides a mechanism which can be used to validate the model with respect to experimental evidence. ROC analysis and other statistical tests were carried out to evaluate the predictive ability of the model and to compare the performance with a state of the art FBA model (iND750); and to evaluate the effect of increasing the coverage of the model from iFF708. The results show that using a logical model rather that a FBA based one results in no significant loss of performance, nor is any loss found by enlarging the coverage of the model to include information from KEGG; and that both iND750 and our logical model represent an improvement over random predictions.

## Methods

### Sources for the logical model

The Logical model of Yeast metabolism has been constructed from two main sources:

1) iFF708 – the Genome Scale Metabolic Network produced by Forster *et al *[6]

2) The Kyoto Encyclopaedia of Genes and Genomes (KEGG) [11,12]

iFF708 consists of a set of reactions for which there exists biochemical evidence in *S. cerevisiae *and a set of open reading frames (ORFs) that encode the gene products that catalyse the reactions. The gene products also belong to enzyme classes, defined by the Enzyme Commission. Forster *et al *used online database sources (MIPS [38], KEGG [11,12], SGD [39], Expasy [40] etc) and literature sources to construct the network. Forster *et al *[35] estimate that 1 man year of research activity is required to identify the reactions corresponding to the metabolic network of a single organism. In constructing the network consideration was given to the presence, stoichiometry, co-factors, reversibility and localisation of reactions in *S. cerevisiae*. The model building phase therefore required many iterations and improvements. ORFs and reactions in iFF708 are localised into various compartments corresponding to (some of) the organelles in the yeast cell. There are 2 compartments – 3 if the immediate external environment (e.g. growth medium) is thought of as another compartment. The internal compartments are the cytosol and the mitochondrion. The resulting reaction list included reactions confined to each of the cell compartments and transport reactions across membranes between the external environment and the cytosol and the cytosol and mitochondrion.

Forster *et al *used the flux balance analysis to simulate various phenotypic and metabolic behaviours. These included the models capability to manufacture key precursor metabolites such as the amino acids, and the ATP related costs of synthesising macromolecules and ultimately biomass from precursors. The latter method was used to validate the model: by comparing the predicted results to previous experimental results from aerobic and anaerobic chemostat cultivation, and investigation of the flux distributions and shadow price calculations allowed the reaction list to be corrected (by hand) – so the predictions from the final model were in agreement with the experimental results. iFF708 has also been validated by comparing growth to a list of essential genes (see section 2).

### Components and structure of the logical model

The logical model and iFF708 both include ORFs, enzymes and reactions found to be part of yeast metabolism. An ORF corresponds to the DNA sequence that is removed or replaced to create each knockout mutant. Enzymes types are the Enzyme Commission number used to classify gene products corresponding to a particular enzyme function, and the reactions are the individual chemical transformation steps that, when taken together represent yeast metabolism. In its original form iFF708 treats the relationships between these components as if they corresponded to a single relational database table, resulting in much duplication of components. For example, with isoenzymes, where the gene products from two or more unique ORFs catalyse the same reaction, the reaction will be repeated in the table as many times as there are isoenzymes. This can lead to confusion when defining the various unique entities or components. Adapting iFF708 to a logical model enables the separation of entities and components into many linked database tables, which is a more natural way to represent the components and their relationships. The current logical model represents a first step – the reactions have been placed in a separate table allowing each reaction to be defined uniquely, with links to the ORF/enzyme components that code/catalyse the reaction. However, there remains much work to be done to extend this representation to allow for additional complexities such as multimeric reactions and protein complexes [5]. Table 9 is a catalogue of the various components and relations found in the logical model. The ORF/Enzyme/Reaction relation is the logical equivalent of the single table of iFF708. It can be seen that adding the information from KEGG has added an additional 688 such relationships. The 1,166 ORFs in the logical model include 226 "unknown" ORFs, where reactions for which there are biochemical evidence have been included in iFF708, but the ORF/enzyme information is not yet complete (this explains the total ORF count of 940 used in model validation – section 2).

**Table 9:** Components of the Logical Model

**Component**	**Size**
ORF/Enzyme/Reaction relations	2303
ORFs	1166
EC Classes	541
Reactions	1087
Metabolites	821

### Representation of ORFs, enzymes and reactions

Two sets of relations lie at the core of the logical representation of the metabolic network:

1. orf_fact(ORF,ECNumber,EnzymeClass,GeneName,

   GeneDescription,ReactionNumber).

**Figure 3:**
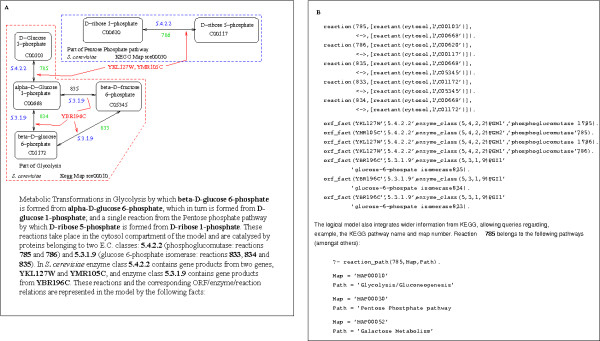
Representation of Metabolic Information: An Example from Glycolysis. **A**: reactions from Glycolysis, **B**: representations in prolog.

This relation corresponds to a single mapping between one ORF, its Enzyme Commission number (also presented as a relation for traversing the hierarchy of EC numbers); the corresponding Gene name; a (brief) description of the function of the gene; and the reaction catalysed by the gene product. The second relation defines the reactions in the logical model:

2. reaction(ReactionNumber,Substrate,Direction,Product).

where *ReactionNumber *is a unique number by which the reaction is identified, *Direction *is either "forwards" (->) or "reversible" (<->) and *Substrate *and *Product *are lists of metabolites together with the cell compartment where the metabolite is found or added to. The stoichiometry of the reaction is also recorded, although the reaction mechanism used to predict the outcome of experiments does not currently make use of this information. Figure 3 uses a simple example from Glycolysis to illustrate the relationships in the logical model. Each Metabolite is stored as a "reactant":

reactant(Compartment,Stiochiometry,CompoundID)

where the *CompoundID *is the unique ID from the KEGG database – where conversion from the notation used in the iFF708 was possible (>90% of metabolites). *Compartment *is one of {external, cytosol, mitochondrion}. The Orf/Enzyme/Reaction relations corresponding to the aber logical model are available as Additional file 1 in the supplementary information to this paper. The reactions are available as Additional file 2.

### The reaction mechanism

The Reaction Mechanism model used to predict the outcome of knockout mutant experiments is essentially the same mechanism as that used in [3]. To relate this model to an observable phenotype we associate the existence of paths through the graph to growth, i.e. if and only if (iff) we can find (deduce) a path from input metabolites in the growth medium to each of a set of essential metabolites (amino acids, the nucleic acid precursors, polysaccharide precursors, lipids, etc.) then the model predicts that the yeast cell will grow indistinguishably from the wild type (observable phenotype). The logical modelling approach is simpler than that of FBA, as it involves the concept of connection (true/false) rather than flux – which removes the requirement to estimate fluxes.

**Figure 4:**
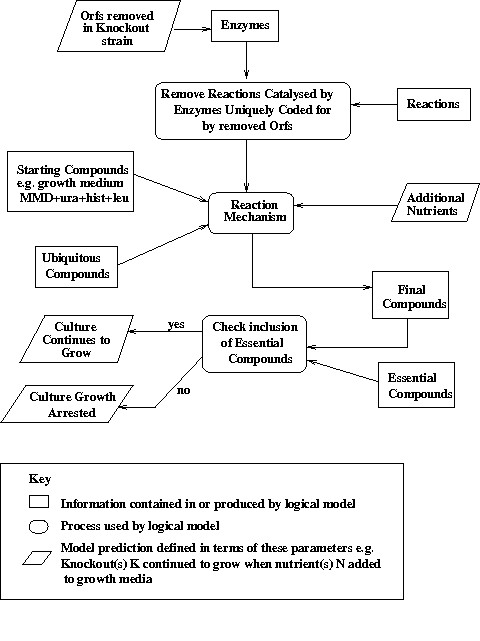
Using The Logical Model to Predict the Outcome of Auxotrophic Experiments In *S. cerevisiae*.

Reactions are processed by checking whether the metabolites on one side of the reaction were present in the cell and adding the metabolites on the other side. Each reaction was processed until no further reactions could be executed and the final complement of cell compounds was used to evaluate the growth outcome for that particular growth experiment. This basic approach has now been altered to incorporate the cell compartments used by the iFF708. KEGG does not specify cell compartments, hence the network components added from KEGG are assumed to be located in the cytosol. Figure 4 illustrates how the reaction mechanism can be used to predict the outcome of the knockout experiments. The inputs to the model points are:

1) The metabolites comprising the minimal growth medium MMD+ura+hist+leu. These are the minimum set of compounds required by the wild yeast for continued growth – as well as the additional nutritional requirements of Uracil, L-Histidine and L-Leucine

2) The ORF(s) knocked out to form the mutant strain

3) Any additional nutrients added to identify possible reactions removed from the metabolic network by knocking out the ORF.

4) Metabolites deemed "ubiquitous" – deemed to be always present in the cell. These include co-factors such as NADPH, NADH, ATP, ADP etc. The full list of ubiquitous compounds is given in Table 10. Ubiquitous compounds are present in very many reactions and the growth simulation algorithm requires their presence in the cell at the start to guarantee the connectivity of the wild-type metabolic network. In biological terms these compounds are present when the yeast cell first separates from the "mother" cell.

**Table 10:** Compounds deemed to be Ubiquitous in *S. cerevisiae*

**Kegg Compound ID**	**Compound Name**
C00005	NADPH
C00004	NADH
C00016	FAD
C00006	NADP+
C00003	NAD+
C00061	FMN
C00575	3'5'-Cyclic AMP
C00399	ubiquinone
C00137	myo-inositol
C00194	coenzyme B12
C00032	protoheme
C00255	riboflavin
C00346	phosphoethanolamine
C00641	1 2-Diacyglycerol
C00448	farnesyl pyrophosphate
C00002	ATP
C00008	ADP
C00342	Thioredoxin
C00007	O2

5) The reactions and ORFs/enzymes defined above

Lists corresponding to the three compartments defined in the iFF708 are created: the lists corresponding to the cytosol and mitochondrion compartments are initially set to contain the minimal medium compounds, and the ubiquitous compounds. The first task of the reaction mechanism is to identify all reactions that can no longer proceed because they are uniquely catalysed by the gene products produced by the ORF(s) removed to create the knockout strain. These reactions are removed from the network before processing by the reaction mechanism proper. The remaining reactions are analysed in turn to see whether the following conditions allowing the reaction to proceed have been met:

1) Irreversible reactions can proceed *iff *(if and only if) all reactants in the substrate are present in the correct cell compartments. When the reaction runs, all reactants found in the product list are added to the compartment specified.

2) Reversible reactions are processed similarly to irreversible reactions, but can also be processed in the reverse direction i.e. *iff *all reactants in the products list are found in the correct compartments, the reactants in the substrate list are added to the specified compartments.

Each reaction is processed in turn and is processed at most once. Currently the logical model simply records the presence or absence of a metabolite and no calculation of the quantities produced is undertaken. This approach is sufficient for the simple continued/arrested growth predictions currently generated, but it will be revised when the model is modified to generate quantitative growth predictions. The mechanism processes all reactions in a cyclical manner as long as 1 or more reactions in the previous cycle have executed. This process ensures that all possible reactions can be processed and as many metabolites as possible are added to each cell compartment.

After processing by the reaction mechanism, determination of continued or arrested growth is made by comparing the final complement of metabolites with a list of compounds deemed essential for healthy cell growth: these include amino acids, nucleic acids, compounds such as D-Glucose 1-phosphate that are essential to the formation of polysaccharides, compounds such as choline that are essential to the formation of membranes, and important intermediary compounds such as pyruvate. The complete list of essential compounds is given in Table 11. The growth status of the simulated experiment is determined as follows:

**Table 11:** Compounds deemed to be essential for healthy growth of *S. cerevisiae*

**Kegg Compound ID**	**Compound Name**	**Compound type/function**
C00041	L-Alanine	amino acid
C00037	Glycine	amino acid
C00079	L-Phenylalanine	amino acid
C00078	L-Tryptophan	amino acid
C00082	L-Tyrosine	amino acid
C00407	L-Isoleucine	amino acid
C00073	L-Methionine	amino acid
C00062	L-Arginine	amino acid
C00049	L-Aspartate	amino acid
C00135	L-Histidine	amino acid
C00097	L-Cysteine	amino acid
C00025	L-Glutamate	amino acid
C00064	L-Glutamine	amino acid
C00047	L-Lysine	amino acid
C00148	L-Proline	amino acid
C00065	L-Serine	amino acid
C00188	L-Threonine	amino acid
C00183	L-Valine	amino acid
C00152	L-Asparagine	amino acid
C00123	L-Leucine	amino acid
C00242	Guanine	base
C00106	Uracil	base
C00147	Adenine	base
C00262	Hypoxanthine	base
C00178	Thymine	base
C00380	Cytosine	base
C00212	Adenosine	nucleoside
C00387	Guanosine	nucleoside
C00294	Inosine	nucleoside
C00214	Thymidine	nucleoside
C00299	Uridine	nucleoside
C00475	Cytidine	nucleoside
C00020	AMP	nucleotide
C00144	GMP	nucleotide
C00130	IMP	nucleotide
C00105	UMP	nucleotide
C00055	CMP	nucleotide
C00002	ATP	energy transfer
C00044	GTP	energy transfer
C00075	UTP	enery transfer
C00005	NADPH	coenzyme
C00004	NADH	coenzyme
C00016	FAD	coenzyme
C00008	ADP	energy transfer
C00035	GDP	energy transfer
C00015	UDP	energy transfer
C00068	Thiamin diphosphate	coenzyme
C03028	Thiamin triphosphate	coenzyme
C00006	NADP+	coenzyme
C00003	NAD+	coenzyme
C00061	FMN	coenzyme
C00575	3' 5'-Cyclic AMP	energy transfer
C00002	ATP	nucleic acid
C00044	GTP	nucleic acid
C00063	CTP	nucleic acid
C00075	UTP	nucleic acid
C00131	dATP	nucleic acid
C00286	dGTP	nucleic acid
C00458	dCTP	nucleic acid
C00459	dTTP	nucleic acid
C00103	D-Glucose 1-phosphate	polysaccharide
C00043	UDP-N-acetyl-D-glucosamine	polysaccharide
C00096	GDPmannose	polysaccharide
C00114	choline	membrane
C00157	lecithin	membrane
C00416	phosphatidate	membrane
C00422	triacylglycerol	membrane
C01694	ergosterol	membrane
C00189	ethanolamine	membrane
C00116	glycerol	membrane
C00137	inositol	membrane
C01120	sphinganine 1-phosphate	membrane
C00668	alphaD-glucose 6-phosphate	intermediate
C00022	pyruvate	intermediate
C00024	acetyl-CoA	intermediate
C00356	3-Hydroxy-3-methyl-glutaryl CoA	intermediate

1) Continued growth is predicted *iff *∀(essential compounds are present in the Cytosol compartment of the model)

2) Arrested growth is predicted *if *∃(essential compound missing from the Cytosol compartment)

Growth of the wild variant with no additional nutrients can be predicted by giving a knockout value of "none" and an empty list for the additional nutrients. To create a usable logical model it was crucial that simulation of the wild variant resulted in continued growth, i.e. the logical model must contain all the reactions required for all of the essential compounds to be added to the cell, and the ubiquitous compounds to contain all the co-factors required by the reactions. *However, including components only found in the iFF708 was not sufficient to predict growth as the network did not include enough reactions to enable synthesis of all the compounds deemed to be essential*. However, this is because the aber model is focussed on modelling the viability of metabolism. This means that our end-points are metabolites considered to be essential for life. The end-points of a flux balance analysis model are metabolites that are important in cell biomass. The two sets of end-point metabolite are therefore not necessarily the same. For example, it would seem clear that a cell must be able to synthesise the immediate nucleotide precursors of RNA and DNA, but these are not all end-points in the iFF708 model. Inclusion of the knowledge representing additions to yeast biology in the KEGG database and added subsequent to the completion of iFF708; as well as an additional reaction, also from KEGG, not yet documented in yeast, but essential for the production of Palmitate, allowed the graph traversal algorithm to reach all essential compounds. (Note – the "palmitate reaction" has now been found to occur in yeast, KEGG [11,12]). Construction of the model was complete when all reactions necessary to simulate continued growth for the wild variant had been added to the model.

### Description of KEGG components used to augment IFF708

Table 12 shows the numbers of ORFs and reactions belonging to KEGG pathways for those ORF/enzyme/reaction relations used to augment the IFF708 model, as well as those reactions and relations found in KEGG that have no pathway assigned. The reactions and relations added from KEGG were inferred from a translation of the LIGAND component of KEGG, where reactions corresponding to ORF/Enzyme relationships not found in IFF708 were collected. (These are the 247 reactions discussed in section 4). This augmentation of iFF708 was undertaken when it was found that the reactions in iFF708 were insufficient to synthesise all of the essential compounds listed in Table 11. There are 318 ORFs involved in the 688 additional ORF/enzyme/reaction relations; of these 55 are also part of iFF708. 37 ORFs have reactions added from KEGG that have no reactions defined in iFF708, i.e. the ORFs had a known EC number but the reaction corresponding to the EC number became part of biological knowledge subsequent to the completion of IFF708.

**Table 12:** KEGG Pathways and total numbers of ORFs and reactions added to the aber model from KEGG and not included in IFF708

**Kegg Pathway**	**Number of Reactions**	**Number of ORFs**
Citrate cycle (TCA cycle)	8	12
Tryptophan metabolism	5	2
Bile acid biosynthesis	1	2
Fatty acid biosynthesis (path 1)	32	1
One carbon pool by folate	1	1
Biotin metabolism	5	1
Selenoamino acid metabolism	10	3
Folate biosynthesis	2	1
Purine metabolism	33	71
Pentose phosphate pathway	1	1
Nicotinate and nicotinamide metabolism	2	1
Fatty acid metabolism	4	1
Lysine degradation	7	2
Propanoate metabolism	1	1
Glycine, serine and threonine metabolism	17	12
C5-Branched dibasic acid metabolism	6	2
Pyruvate metabolism	8	5
Methionine metabolism	10	3
Alanine and aspartate metabolism	4	4
Ubiquitin mediated proteolysis	1	14
Sulfur metabolism	8	1
Nitrogen metabolism	2	2
Glutamate metabolism	4	4
Aminosugars metabolism	2	1
N-Glycans biosynthesis	8	14
Starch and sucrose metabolism	3	4
Pentose and glucuronate interconversions	2	1
Glycerolipid metabolism	10	3
gamma-Hexachlorocyclohexane degradation	7	7
Phenylalanine, tyrosine and tryptophan biosynthesis	2	5
No Pathway Annotation	50	153
RNA polymerase	5	31
Lysine biosynthesis	5	1
Oxidative phosphorylation	3	26
Butanoate metabolism	10	5
Fructose and mannose metabolism	1	1
Pyrimidine metabolism	29	68
1,4-Dichlorobenzene degradation	3	1
Phosphatidylinositol signaling system	1	10
DNA polymerase	5	18
Porphyrin and chlorophyll metabolism	2	1
Arginine and proline metabolism	1	2
Cysteine metabolism	11	3
Galactose metabolism	1	1
Pantothenate and CoA biosynthesis	6	2
Aminoacyl-tRNA biosynthesis	18	27
Glycolysis / Gluconeogenesis	12	8
Valine, leucine and isoleucine biosynthesis	13	10
Riboflavin metabolism	5	7

The relations corresponding to the 247 additional reactions belong to 47 pathways described in KEGG, however the largest proportion of these relations correspond to reactions that are not included in KEGG pathways. Purine metabolism (33 reactions), Fatty acid biosynthesis (path 1) (32 reactions) and Pyrimidine metabolism (29 reactions) have the largest numbers of reaction assignments; Purine metabolism (71 ORFs), Pyrimidine metabolism (68 ORFs) and RNA polymerase (31 ORFs) have the largest numbers of ORF assignments. There is no simple relationship between the numbers of ORFs in a pathway and the number of reactions, e.g. the 32 new reactions added to the Fatty acid biosynthesis (path 1) pathway are all coded for by a single ORF.

### Model validation

Model validation [41] is a vital stage in the construction of any model. This can be done either by 1) direct experimentation where the model also determines the most relevant experiments (preferably), or 2) by comparison with existing datasets. The former method is intrinsic to methods of system identification, where models are either generated or improved by machine learning/statistics techniques e.g. equation discovery and computational scientific discovery.

It is possible to use the second method to obtain some measurements of the validity of metabolic models of yeast by comparing the predicted outcomes of auxotrophy experiments to the results of a whole genome gene-deletion study undertaken by Giaever *et al *[4]. This study involved two repeats of competitive batch experiments where all knockout strains were grown on an agar base supplemented by a number of both defined and undefined growth media. Measurements of growth were obtained after 5 and 15 generations and are presented as fitness comparisons against the growth of the average strain on rich undefined medium (YPD – Yeast Extract, Peptone and Dextrose), where strains growing slower than average are defined as *sensitive *and those growing faster as *refractory*. Growth data for each particular strain corresponds to the relative abundance of strain-specific DNA tags found by hybridisation to a custom high density oligonucleotide array, and take the form of continuously valued relative fitness scores, which are expressed as "units of deleterious growth" [4]. There are 4 sensitive/refractory measurements for each strain/growth medium combination. Giaever *et al *used a sensitivity score cut off of >20 units of deleterious growth to identify strains with a significant deleterious phenotype after 5 generations, rising to >100 for 15 generations, reflecting the observation that strains found to be sensitive earlier would represent genes that are more important for cell growth. Giaever *et al *[4] also compiled a list of *essential *strains that did not grow on YPD. Model validation therefore assessed the models' performance on predictions of gene essentiality as well as on a more comprehensive analysis of growth predictions which combine the essential gene listings with the results from the most relevant defined medium. This was a minimal medium (MMD+ura+hist+leu), which contains the minimum sets of compounds required for wild type growth as well as additional nutritional requirements for uracil, histidine and leucine.

## Authors' contributions

RDK initiated the logical modelling concept and constructed the prototype model. KW constructed the logical model and carried out the validation experiments and the statistical tests. RDK and KW wrote and revised the manuscript.

## Supplementary Material

Additional file 1 ORF, Enzyme and Reaction Relations. This file contains Prolog facts corresponding to the relationships between ORFs, Enzyme Commission Numbers and Reactions for the Logical Model of Yeast Metabolism. Click here for file

Additional file 2 Reactions. This file contains Prolog facts corresponding to Substrate and Product information for Reactions in the Logical Model of Yeast Metabolism. Click here for file
